# Influence of glycerol and an alternative humectant on the immediate and 3-hours bactericidal efficacies of two isopropanol-based antiseptics in laboratory experiments in vivo according to EN 12791

**DOI:** 10.1186/s13756-017-0229-5

**Published:** 2017-06-27

**Authors:** Miranda Suchomel, Martina Weinlich, Michael Kundi

**Affiliations:** 1 Institute of Hygiene and Applied Immunology, Medical University of Vienna, Kinderspitalgasse 15, 1090 Vienna, Austria; 2 Center for Public Health, Medical University of Vienna, Kinderspitalgasse 15, 1090 Vienna, Austria

**Keywords:** Isopropanol-based, Surgical hand rub, Antiseptics, Humectant

## Abstract

**Background:**

Guidelines for hand hygiene recommend the use of alcohol-based hand rubs containing humectants in order to improve dermal tolerance. However, the bactericidal efficacy of pre-surgical hand rubs is negatively affected by the WHO-recommended humectant glycerol, especially the 3-h efficacy. The aim of this study was to investigate whether replacing glycerol as humectant increases the bactericidal efficacy of surgical hand rubs based on isopropanol (75%, wt/wt).

**Material and methods:**

The efficacy of 3 and 5 min applications of a modified WHO II-formulation (containing lower glycerol concentrations) and the TPH 5766 hand rub which contains a new humectant (containing ethylhexylglycerin, dexpanthenol and a fatty alcohol) were compared to the European Norm 12,791 reference (n-propanol, 60%, vol/vol) immediately following and 3 h after application.

**Results:**

Immediately after application both isopropanol-based surgical rubs approximated the performance of the reference. The 3-h effect of the modified WHO II-formulation was found to be less efficacious than the EN 12791, showing a 30% decrease in log_10_ reduction values. The 3-h post application effect for the TPH 5766 hand rub was found to not be different from EN 12791.

**Conclusion:**

Based on our data, the bactericidal efficacy of isopropanol-based surgical hand rubs can best be obtained if glycerol is not used in the formulation. Unlike glycerol, a humectant comprised of ethylhexylglycerin, dexpanthenol and a fatty alcohol was found not to decrease hand rub effectiveness. Further investigation of the bactericidal efficacy of other humectants is necessary and may prove useful.

## Background

Pre-surgical hand preparation is an accepted infection control standard worldwide with the aim of eliminating the pathogenic bacteria and reducing the resident flora on the hands of the surgical team to a minimum [1]. Guidelines for hand hygiene as published by the Centers of Disease Control and Prevention [1] and by the World Health Organization [2] recommend alcohol-based rubs as the primary method for hand hygiene. Furthermore, both guidelines recommend that alcohol-based rubs should contain humectants in order to improve dermal tolerance. For this, the two WHO-recommended ethanol- or isopropanol-based formulations for hygienic and pre-surgical hand antisepsis in developing countries (WHO I and WHO II) contain glycerol as a humectant. As previously shown [3], both original WHO-formulations do not meet the efficacy requirements of the European Norm (EN) 12,791 [4], the in vivo laboratory assay for testing the bactericidal efficacy of pre-surgical hand preparations. Increasing their alcohol concentrations by 5% and halving the glycerol concentration brought both WHO formulations in compliance with EN 12791 [5]. In this study, we compared the bactericidal performance of n-propanol (60% vol/vol) as a reference treatment prescribed by EN 12791 [4] with two formulations in different applications (3 and 5 min). Firstly, we tested the modified WHO-formulation II [5] with a glycerol concentration of 0.725%. Secondly, we used a new hand rub, TPH 5766, also based on isopropanol (75%, wt/wt), containing an innovative humectant (ethylhexylglycerin, dexpanthenol combined with a fatty alcohol) without glycerol at 1.60%. The aim was to investigate whether reduction or replacement of glycerol by another humectant leads to an acceptable efficacy as pre-surgical hand rub according to EN 12791, with emphasis on its effectiveness 3-h after application.

## Material and methods

Formulations used in this study; (i) WHO II-modified: isopropanol (pro analysi, Merck, Germany) 75% (wt/wt); hydrogen peroxide (pro analysi, Merck) 0.125% (vol/vol); glycerol (pro analysi, Merck) 0.725% (wt/wt); (ii) TPH 5766 (desmanol pure®; Schülke & Mayr GmbH, Germany) containing isopropanol 75% (wt/wt); a humectant at 1.60% (wt/wt) consisting of ethylhexylglycerin, dexpanthenol and a fatty alcohol; (iii) n-propan (pro analysi, Merck) 60% (vol/vol), the reference as required by EN 12791.

Sampling and dilution fluids were tryptic soy broth, TSB (Caso broth®, Merck). Counting plates used tryptic soy agar, TSA (Caso agar®, Merck). Non-medicated, sterile soft soap (APOCA, Austria) was used for preparatory hand wash. Neutralizing agents were not necessary because dilution with pure broth was shown in previous validation tests to neutralize any antimicrobial effect for each of the tested formulations.

The bactericidal activities of the two study formulations applied for 3 and 5 min were compared with that of the reference of EN 12791.

The study was conducted at the Institute for Hygiene and Applied Immunology, Medical University, Vienna, Austria. The laboratory was accredited according to EN ISO/IEC 17025:2005 [6] and recognized by the national accreditation body “Akkreditierung Austria”. All areas of testing were approved and reported to the Federal Ministry of Science, Research and Economy, Austria. This study was performed in compliance with the World Medical Association, Declaration of Helsinki - Ethical Principles for Medical Research Involving Human Subjects [7]. Ethics committee approval was not required because in accordance to the EN 12791 methodology [4] the tests were conducted on clean, not artificially contaminated hands of volunteers and the study formulations were commonly used preparations for hand antisepsis in health care. Twenty persons, all employees of the Institute for Hygiene and Applied Immunology, Medical University, Vienna, Austria, participated as volunteers. The participation was voluntarily with the possibility to withdraw without giving any reason and without personal disadvantages. No identifying information was collected from the volunteers and all had given their written informed consent.

A balanced design was used with 5 experimental groups, each of 4 volunteers, differing in the sequence of the 5 experimental conditions (two formulations, two application durations (3 and 5 min), plus the reference). Between each run an interval of 1 week was kept to allow regrowth of the normal skin flora. At the end of the fifth test run, every subject had used each treatment once.

The test method followed EN 12791 [4]: After a preparatory hand wash for 1 min with 5 mL of non-medicated soap pre-treatment values were established. For this purpose, the fingertips, including the thumbs, of both hands were rubbed and kneaded for 1 min at the bottom of a Petri dish (Ø 9 cm) - one for each hand - each containing 10 mL of TSB. Next, one of the disinfection procedures was performed. Portions of 3 mL of the respective formulation were poured into the cupped dry hands and rubbed onto both hands up to the wrists. As many portions (between 3 and 6 applications) were applied as were necessary to keep hands wet for the preset length of surgical hand rubs. Immediately after antisepsis, the fingertips of one randomly selected hand were sampled for the assessment of the immediate post-treatment values as described above. The other hand was gloved with a sterile surgical glove (simulating a surgery) and sampled after 3 h for the assessment of the 3-h post-treatment values as described in the Norm. From all sample fluids dilutions of 10–1 and 10–2 were prepared in TSB. An aliquot of 0.1 mL from each dilution was then spread onto TSA with sterile glass spatulas. Counting plates were incubated, colony forming units (cfu) were counted and the number of cfu per milliliter was calculated. Pre- and post-treatment counts were transformed into decimal logarithms and reduction factors (log10 RF) were calculated, separately for immediate and 3-h effects.

Although EN 12791 schedules Wilcoxon-Wilcox tests we have chosen analysis of variance (ANOVA) and a priori linear contrasts for their superior statistical power to test the mean log10 RF values for significant differences. Following the provisions of EN 12791, significant differences between the means obtained with the test formulations and the reference were supposed if respective P-levels were ≤0.1 (one-sided) for immediate and *P* ≤ 0.05 (two-sided) for 3- h effects. To evaluate differences in the bacterial reduction capability of the formulations and between durations of application two-factor ANOVAs for repeated measures were performed with the differences of the respective log10 RF component of interest minus the log10 RF of the corresponding values of the reference.

## Results

The immediate effects are shown in Table 1. The immediate effectiveness of the 3 min-application of the modified WHO II-formulation was slightly less but not significantly different from that of the reference preparation. When used for 5 min WHO II-modified was slightly more efficacious. Neither was statistically significant. Comparably, the TPH 5766 hand rub had comparable efficacy as efficacious as the reference when applied for 3 min; after 5 min its effect was somewhat higher, though not significantly.

**Table 1:** Immediate bactericidal effects of the modified WHO-formulation II and TPH 5766 as compared to that of the reference hand rub for pre-surgical hand antisepsis according to EN 12791

Formulation	duration of application (*n* × 3 ml)	Mean (*n* = 20) log_10_ pre-value	Mean (*n* = 20) log_10_ reduction	*p*-value for difference to reference
Reference	3 min	4.46 ± 0.63	2.56 ± 1.10	
WHO II-modified	3 min	4.36 ± 0.52	2.22 ± 1.15	0.176
WHO II-modified	5 min	4.45 ± 0.44	2.65 ± 1.03	0.731
TPH 5766	3 min	4.41 ± 0.64	2.53 ± 1.20	0.887
TPH 5766	5 min	4.37 ± 0.52	2.75 ± 1.25	0.513

*P* < 0.1, one-sided (ANOVA, linear contrasts)

Thus, the immediate effect of both formulations complied with the requirements of EN 12791.

Table 2 summarizes the 3-h results. Whereas WHO II-modified did not comply with the requirement of EN 12791 when used for 3 min, it did so after 5 min of application. The performance of TPH 5766 met the requirement for both durations. Neither the different preparations (*P* = 0.317) nor durations of application (*P* = 0.079) were statistically significant for the immediate effect. At 3-h post application, the type of preparation had a significant (*P* = 0.004) difference in observed efficacy, but not so the duration of application (*P* = 0.289). TPH was significantly superior over modified WHO II-formulation (*P* = 0.024) for the 3-min post application observations. With an application of 5 min the effect of WHO II-modified was not significantly (*P* = 0.123) less efficacious.

**Table 2:** Three-hours bactericidal effects of the modified WHO-formulation II and TPH 5766 as compared to that of the reference hand rub for pre-surgical hand antisepsis according to EN 12791

Formulation	duration of application (*n* × 3 ml)	Mean (*n* = 20) log_10_ pre-value	Mean (*n* = 20) log_10_ reduction	*p*-value fordifference to reference
Reference	3 min	4.47 ± 0.55	2.03 ± 0.93	
WHO II-modified	3 min	4.30 ± 0.59	1.41 ± 1.32	0.014^a^
WHO II-modified	5 min	4.51 ± 0.58	1.74 ± 1.15	0.081
TPH 5766	3 min	4.41 ± 0.65	2.08 ± 1.24	0.847
TPH 5766	5 min	4.32 ± 0.65	2.13 ± 1.17	0.651

*P* < 0.05, two-sided (ANOVA, linear contrasts)

^a^significantly less efficacious than the reference

Because the different humectants may lead to differences in volumes applied which could lead to differences in efficacy, we have compared the actual volumes applied by the volunteers. There were no significant differences for the 3 (*P* = 0.781) or the 5 min (*P* = 1.0) applications.

## Discussion

In order to compare these results with those of other tests according to EN 12791 we followed its protocol. Therefore, the results of this study are those encountered of controlled in vivo laboratory experiments with volunteers. They demonstrate the influence of glycerol and another humectant on the efficacy of pre-surgical hand antiseptics under standardized pre-set conditions which may not necessarily reflect those during clinical use.

Due to their strong and rapid microbicidal activity, alcohol-based hand rubs are recommended in hand hygiene in guidelines [1, 2]. Alcohols are generally well tolerated but frequent use of alcohols can cause skin problems. Therefore, humectants are commonly added to alcohol-based rubs [8]. In commercial preparations, glycerol is often used and its positive effect on skin condition and user acceptability has been demonstrated [9]. However, the bactericidal efficacy of alcohol-based surgical rubs is negatively affected by glycerol, especially their 3-h effect [10]. Barbadoro et al. compared the efficacy of an alcohol-based hand rub containing glycerol with products based on chlorhexidine and povidone-iodine [11]. They observed that the best results were achieved with the alcohol. Hand rubs with glycerol formed sticky agglomerates - presumably formed by the reaction between flaking skin cells and glycerol - on 35% of volunteer hands, which resulted in lower bacterial reductions compared with volunteers who did not show this phenomenon. Minimizing the glycerol content or using alternative humectants has been speculated to possibly increase the bactericidal effect of alcohol-based hand rubs [10]. Therefore, in this study, the efficacies of the WHO II-modified formulation with a lower glycerol concentration and of a new formulation, TPH 5766, also containing 75% isopropanol combined with another humectant consisting of ethylhexylglycerin, dexpanthenol and a fatty alcohol, were compared according to EN 12791. And, indeed, TPH 5766 containing the same alcohol at the same concentration but with another humectant proved to exert a comparable or even better bactericidal activity than modified WHO II-formulation. When testing hand disinfectants according to EN 12791 the release of skin flora is assessed before, immediately and 3 h after antisepsis. The finding that reducing or replacing glycerol as an emollient leads to a stronger reduction of this flora is plausible when one considers that the main-part of the predominant flora of the skin microbiome is able to ferment glycerol, and, therefore, to use it as a growth factor. This means that with glycerol, surviving skin flora will grow faster within the 3 h measuring period than without glycerol, hence producing an undesired higher 3-h value.

This is indirectly confirmed by our finding that TPH 5766 was more efficacious than the glycerol containing formulation.

## Conclusion

Based on these data, inhibition of skin flora and therefore increased bactericidal efficacy of isopropanol-based surgical hand rubs can best be obtained if glycerol is used in a minimal but still skin-protective concentration or replaced by other chemicals. Further, emollients need to have a minimal risk for undesired local or systemic effects. Therefore, controlled testing under clinical conditions for undesired adverse reactions and degree of acceptance by health care workers is, nevertheless, still needed. Also, costs of a new formulation have to be considered, especially in the Third World for which, in fact, the WHO-recommended formulations have been developed.
